# A Sustainability Model for Family-Focused Practice in Adult Mental Health Services

**DOI:** 10.3389/fpsyt.2021.761889

**Published:** 2022-01-18

**Authors:** Becca Allchin, Bente M. Weimand, Brendan O'Hanlon, Melinda Goodyear

**Affiliations:** ^1^School of Rural Health, Monash University, Melbourne, VIC, Australia; ^2^Eastern Health, Mental Health Program, Melbourne, VIC, Australia; ^3^Division Mental Health Services, Akershus University Hospital, Lørenskog, Norway; ^4^Faculty of Health and Social Sciences, Department of Health, Social and Welfare Studies, University of South-Eastern Norway, Drammen, Norway; ^5^The Bouverie Centre, La Trobe University, Melbourne, VIC, Australia; ^6^Emerging Minds, Hilton, SA, Australia

**Keywords:** sustainability, family-focused practice, mental health promotion, parents, mental ill-health, Let's Talk about Children intervention

## Abstract

**Background:**

Translating evidence-based practice to routine care is known to take significant time and effort. While many evidenced-based family-focused practices have been developed and piloted in the last 30 years, there is little evidence of sustained practice in Adult Mental Health Services. Moreover, many barriers have been identified at both the practitioner and organizational level, however sustainability of practice change is little understood. What is clear, is that sustained use of a new practice is dependent on more than individual practitioners' practice.

**Design and Method:**

Drawing on research on sustaining Let's Talk about Children in adult mental health services and in the field of implementation science, this article proposes a model for sustaining family focused practice in adult mental health services.

**Sustainability Model for Family-Focused Practice:**

An operational model developed from key elements for sustaining Let's Talk about Children identifies six action points for Adult Mental Health Services and their contexts to support the sustainability of family-focused practices. The model aims to support Services to take action in the complexity of real-world sustainability, providing action points for engaging with service users and practitioners, aligning intra-organizational activities, and the wider context.

**Conclusion:**

The model for sustaining family-focused practice draws attention to the importance of sustainability in this field. It provides a practical framework for program developers, implementers, adult mental health services and policy-makers to consider both the components that support the sustainability and their interconnection. The model could be built on to develop implementation guides and measures to support its application.

## Introduction

Research in the past 30 years has explored the impact of a parent's mental ill-health on family life, raising awareness of the importance of family-focused practices for parents and their children (1–6). Such work in mental health services identifies a dual focus (i) improving the outcome for the person with the mental illness and (ii) reducing distress in family members while building their resilience and well-being (7–9). In Adult Mental Health Services (AMHS), family-focused practices encompass approaches, programs, interventions, models and frameworks that acknowledge the whole family context of the person receiving services (2, 10). These take into account the relational nature of recovery and therefore attend to the person's parenting role and family relationships and provide support to the parent in the context of their children and family, while also attend to the intergenerational mental health needs (10–12). Components of effective interventions include psychoeducation directed at both parents and children, adapting parenting behavior through increasing parent agency and skill building, and improving family communication particularly about mental illness (13).

There is now established evidence that these family-focused practices have an impact on supporting the parent in their parenting role and their mental health recovery (10, 14–17) and on protecting children and promoting their resilience (18–21). There are now many evidence-based family-focused practices or programs and documentation of ongoing delivery of programs (22–24). There is, however, little evidence of the use family-focused practices in routine care within AMHS (25–30).

To understand the lack of use of evidence-based family-focused practice in AMHS, research efforts have explored barriers at the practitioner and organization level. Inadequate family-focused training has been identified at the practitioner level, as has a lack of the necessary knowledge, skills and confidence in family-focused practice, limiting their ability to identify and support the parenting role of their clients while also holding their clients' children in mind (31–39). These barriers are reinforced by organizational contexts that do not routinely identify their client's parental status (29, 40–42) and are funded to work with individuals within a biomedical professional-centered approach that is focused on treatment in acute episodic care (10, 11, 20, 43). The formalized, centralized organizational structures common in AMHS are also known to foster the continuation of existing cultures, making innovation and change more difficult (44). These shape the work and the workforce to make it difficult to prioritize working with whole families with the preventive and early intervention approach inherent in family-focused practices in under-funded settings (2, 43, 45). Additionally, a lack of government and organizational structures such as policies and directives, create an authorizing void for the promotion of family-focused practices and impede leadership support for translating such practices into practitioner's everyday work (45–48).

In recent years, greater attention to the process of implementing family-focused practices has resulted in developments to address these barriers. These include practice guidelines and frameworks for family-focused practice in AMHS (19, 49, 50), integrated training, implementation and research programs (51–53) and international collaboration supporting the integration of policy and research (54–56). While these significantly contribute to the understanding of what is needed to sustain family-focused practice in AMHS, there is a need to draw this knowledge together to consider the multiple components in combination to assist AMHS to implement and sustain family-focused practice. This article proposes a model for sustaining family-focused practice in AMHS.

## Design and Method

The barriers to family-focused practice noted above illustrate the multi layered factors that impact sustainability and show it to be intimately linked with implementation. While sustainability is focused on the degree to which the intervention can continue to deliver its planned benefits, it relies on practitioners who are able to faithfully deliver it, who in turn need support from their organizations to enable them to deliver its core elements (57).

The field of implementation science studies strategies and structures to support implementation of research into practice and has developed a growing body of frameworks, models and theories (58, 59). It has been posited, however, that much of the work developed in implementation science is used to support other researchers but is not yet common knowledge within the practice world (60). Acknowledging healthcare settings as complex entities, has additionally led to a call for integrating complexity science with implementation science to enable a more dynamic approach to implementation research and practice that fits the reality of change in healthcare setting (61–63).

Sustaining family-focused practice is the work of the healthcare setting. While researchers, purveyors or innovators may develop, trial, pilot, or even implement a family-focused practice, the ongoing work of sustainability is dependent on those within the healthcare setting making the ongoing adjustments necessary for the practice to be ongoingly delivered (57, 64, 65). Equipping healthcare services to apply implementation science knowledge could assist them with evidence-based strategies for applying the necessary adjustments locally. This, however, requires the development and application of implementation tools, described by Westerlund et al. (60) as user- or practice-friendly tools, that are suitable for the context and flexible and able to be adapted to fit settings.

A model is an intentional simplification that can provide an accessible description to guide an implementation process or investigation and so can be applying theory to practice (58). Building on what is known about practitioner and organizational barriers to family-focused practice and frameworks from implementation science, this article proposes a model for actions to support the sustaining a family-focused practice in AMHS.

The model is drawn from a series of five mixed method studies exploring the sustainability of the family-focused practice, Let's Talk about Children (LTC) in eight AMHS in Victoria, Australia, involved in a RCT of LTC (52). The series of studies documented practitioner use and organizational capacity in the eight AMHS and developed an explanatory model of factors enabling sustainability in one AMHS (45, 66–69). The research series used a participatory research approach working in partnership with change agents within AMHS across Victoria. This helped to ground the model in practice wisdom and supporting it to be what Westerlund et al. (60) describes as “practice-friendly.” The outcomes of these studies were clustered deductively using sustainability and implementation models and frameworks (65, 70, 71). Five clusters of key elements were identified as influencing LTC's sustainability (69). These clusters related to (1) the parent, (2) the practitioner, (3) the organization, (4) the wider context and (5) the implementation context (see Table 1). While these elements can be considered individually, the studies' outcomes highlight the intersectionality between these elements as an important contributor to sustainability.

**Table 1:** Key elements influencing sustainability of LTC.

**Cluster of influences**	**Influencing elements**
Parent	Parent identification data Parent trust/connection with practitioner
Practitioner	Access to parents on caseload Adapt LTC to parent-consumers needs and working model of team Use of practice support where available Team's workload Characteristics (gender, profession, prior experience) Practitioner's use of LTC monitored Practitioner connection with parent
Organizational	Organizational ownership of implementation Senior leadership communicating priority Middle management enabling fit to everyday work Feedback loops connecting data collections Organizational structures • Allocation system accounting for parenting role • Practitioner training and support infrastructure • Data collection systems -parent numbers, trained practitioners, practitioner's application post training • Reporting systems that consider parent, child and family well-being • Organizational adjustments to fit LTC
Wider Context	Introduction of recovery-oriented policy Parent, child and family focused Mental Health Act Government funded family-focused service development positions in AMHS
Implementation context	Research trial with trusted organizations Supported localized implementation Internal implementers within AMHS Parallel innovations - free online training and resources package

For example, a parent cannot be offered the family-focused practice if the practitioner allocated to them is not equipped with the skill and confidence to use it. Without a system to identify clients as parents, skilled practitioners may not be allocated parents. A skilled practitioner will find it difficult to maintain confidence if they are only rarely allocated a parent. Without a monitoring system, there will be no way of knowing if a practitioner is applying their skills, and if parents are being offered the family-focused practice to know if is being sustained. Additionally, without monitoring there is nothing to inform decision making and provide input for troubleshooting difficulties. If the wider systems do not fund AMHS to work with families or prioritize preventative mental health, an organization may find it difficult to integrate the family-focused practice into their model of care.

Conversely, a training program does not ensure sustainability, as trained practitioners may not be able to implement their new skills in practice. A system for identifying the parental status of clients will, in itself, not ensure that they are allocated for their care to trained practitioners, or have practitioners who are endorsed with the time and scope to use their skills. Data collected without feedback loops to adjust implementation cannot inform policy, training, support and allocation structures. These are each part of the picture of sustainability but on their own will not enable sustainability. They are required to be applied in combination.

## Sustainability Model for Family-Focused Practice

**Figure 1:**
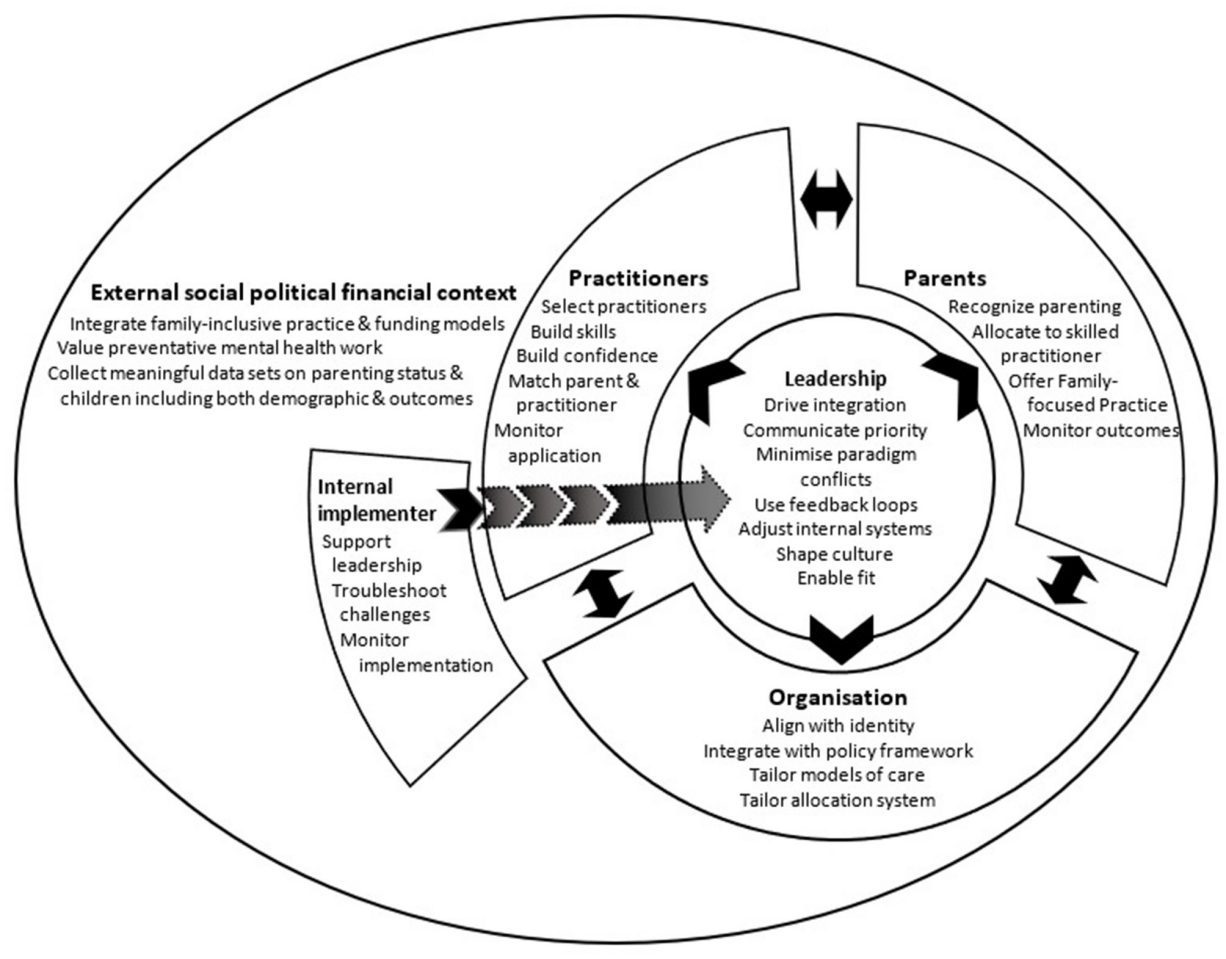
Sustainability model for family-focused practice.

Working from these known key elements influencing sustainability of the family-focused practice of LTC, the following model was developed to operationalize the action points for AMHS and their external contexts to support family focused practice practice more broadly (See Figure 1: Sustainability model for family-focused practice). Framed in outcomes focused language to help operationalize action and reflecting the interconnecting nature of the elements, the model identifies six points of meso (intra organizational) and macro (broader contextual) level action, each incorporating multiple elements. Designed as an intentional simplification for a practical purpose, this model aims to support AMHS to hold in mind the complexity of sustainability and the requirement of simultaneous actions while providing actionable starting places. The first two actions points relate to how the AMHS engages with its service users and their practitioners. The next three action points focus on internal organizational activities important for implementation and sustainability. The last action point articulates important actions in the wider context.

### Recognize, Allocate, and Measure Outcomes for Parents, Children, and Families

Recognition of a client's parental status can allow for service delivery to be tailored to address their, their children's and their family's needs. Knowledge of prevalence of parenting amongst the organization's clients can be used to drive the number and location of skilled practitioners needed to adequately enable parents, children and families to access family-focused practice. Organizations can support parents by allocating them to practitioners with the skills and confidence to deliver family-focused practice. Flexible allocation systems that can attend to the match between parent and practitioner readiness can support the therapeutic alliance and enable family-focused practice to be delivered. Recognition of parenting status also can support the organization's capacity to apply appropriate measures that assist them in monitoring both what services are delivered and if they give the expected benefits for parents, children and families.

### Select, Support, and Monitor Practitioners

The selection of practitioners needs to take into account factors such as access to parents on their caseload, the practitioners' skills and knowledge of the impact of mental illness on parents, children and families, as well as their ability to hold a dual perspective while working with an individual. Building practitioners' skills and confidence to use family-focused practice requires flexible practice support that facilitates their capacity to reflect on and monitor their own practice against expected outcomes. Such support structures need to be co-developed so as to be tailored to fit practitioners' specific needs. Developing systems to monitor practitioners' application of family-focused practice provides a feedback loop that can help to identify support needs, communicate priority and address fidelity issues.

### Integrate Within Organization Identity and Structures

Aligning family-focused practice within an organization's identity and integrating it into policy structures, can enable models of care to be tailored to fit family-focused practice, and support the incorporation of its core competencies into position descriptions and recruitment processes. Embedding family-focused practice into organizational policy also supports the development of infrastructure to enable practice, such as practitioner training, support and monitoring systems, and parent recognition and allocation systems. Organizational policy can additionally provide an anchor for family-focused practice in times of personnel or structural change that can facilitate its continued use. Furthermore, integrating outcome measures and reporting structures that incorporate whole-of-family well-being can help to reinforce a preventative mental health focus that is foundational to family-focused practice.

### Leadership to Drive Sustainability

Organizational ownership is needed to support the internal adjustments required for the integration and sustainability of family-focused practice in AMHS. Adjustments to complex, internal structures need whole-of-organization commitment that requires leadership at multiple levels within the organization. At a higher level this includes communicating this work as a priority, developing training and support infrastructure, creating feedback loops and reporting systems. At the level of middle management this includes building cultures that promote recovery-focused family-inclusive mental health practice, facilitating the translation of family-focused practice into everyday practice and utilizing the feedback loops to support practice. Held together, the multiple levels of leadership and the structures they provide can help to minimize paradigm conflicts that exist for family-focused practice in AMHS.

### Local Support for Implementation and Sustainability

Having an internal implementer to support leadership in the implementation process can help support sustainability. The presence of the internal implementer can be an anchor to the priority of the work and provide resources for leadership to build practitioners' skills and confidence. Working with leadership, they can assist in monitoring implementation through feedback loops that can enable ongoing adaptation of implementation processes to support sustainability.

### Incorporate Family-Inclusive Preventative Mental Health Care in the Wider Context

Incorporating a family-inclusive, preventative lens within the funding and political context within which AMHS operates, creates a foundation for sustaining family focused practice. Integrating these lenses into recovery-focused mental health practice can support shifts in the funding models from an individual to whole-of-family perspective and the valuing of preventative mental health work that underpins family-focused practice. These shifts create an authorizing environment for AMHS leadership to give priority for delivering family-focus practice and the integration of family-focused practice into AMHS models of practice. These shifts also reinforce the need for reporting measures that account for parent, children and family outcomes and that emphasize resilience and well-being rather than risk.

## Implications/Application

This model for sustaining family-focused practice in AMHS provides points of action for AMHS and their external contexts. The model extends existing peer reviewed work that identifies barriers and facilitators of implementation and models that explain sustainability, through drawing these together to provide actionable points of focus for those within an adult mental health system. It is intended to provide a practical framework for integrating the evidence in implementation science as applied to family-focused practice. The model is envisioned to be a tool for program developers, implementers, AMHS and policy-makers to consider both the components that support the sustainability and their interconnection.

As noted here, there is a need for ongoing attention to the complexity and importance of sustainability in the field of parents with mental ill-health, and their children and families. As AMHS are complex and changing entities, ongoing attention to the interconnection between practice, and the organisation's capacity to support practice, is required to enable continued quality of care. Sustaining family-focused practice, shifts the focus from the program, innovation or practice being implemented, to the mechanisms that enable them to be able to be utilized beyond the focused implementation or research trial. As sustainability happens within the work of the health service, equipping AMHS to not only implement but also sustain family-focused practice is pivotal for the field to promote better outcomes for parents, children and families.

This model goes some way to assist this process by identifying points of actions for AMHS and their external contexts, that are articulated as part of a whole, in order to address the complexity and work toward sustainability.

Further work is required to develop practice-friendly tools to support the application of this model. Practical implementation guides could operationalize each of the points of action. Monitoring and measuring tools could provide feedback loops on sustainability for AMHS. Coproduction of these application tools would support their usability by AMHS for their specific contexts. Additionally, this model provides a framework for developers of innovations, practices or interventions to build practice-friendly tools to support their sustained use in AMHS.

## Conclusion

The model showcases the importance of actions that need operationalization at the organizational and wider context level to be able to influence the multiple systems involved in creating sustained family focus practice. This level of complexity can be overwhelming and difficult for program developers, implementers, AMHS and policy-makers to hold in mind, leading to a focus on the actions or elements in isolation. The model, however, highlights the inadequacy of an isolated view of actions or elements if the aim is to build sustainability at the local level that fit their context.

## Author's Note

The content in this manuscript has been adapted from BA's thesis available online https://doi.org/10.26180/14214686.v1.

## Data Availability Statement

The original contributions presented in the study are included in the article/supplementary material, further inquiries can be directed to the corresponding author/s.

## Author Contributions

BA conceived the article, developed the concept of the sustainability model, and drafted the article. MG, BO'H, and BW contributed to the interpretation of the analysis, the refining of the manuscript, reviewed drafts, and contributed to the write up. All authors were contributors to each of the five studies that underpin this work. All contributed to the analysis and drawing together of their combined outcomes and refining the sustainability model.

## Funding

An Australian Government Research Training Program Scholarship supported BA in her doctorate of philosophy, during which she developed the body of work underpinning the model.

## Author Disclaimer

This article represents the authors' original work and has not been submitted for publication elsewhere.

## Conflict of Interest

The authors declare that the research was conducted in the absence of any commercial or financial relationships that could be construed as a potential conflict of interest.

## Publisher's Note

All claims expressed in this article are solely those of the authors and do not necessarily represent those of their affiliated organizations, or those of the publisher, the editors and the reviewers. Any product that may be evaluated in this article, or claim that may be made by its manufacturer, is not guaranteed or endorsed by the publisher.
